# miR-423 sponged by lncRNA *NORHA* inhibits granulosa cell apoptosis

**DOI:** 10.1186/s40104-023-00960-y

**Published:** 2023-12-05

**Authors:** Yuqi Li, Zhuofan Zhang, Siqi Wang, Xing Du, Qifa Li

**Affiliations:** College of Animal Science and Technology, Nanjing Agricultural University, Nanjing, 210095 China

**Keywords:** Granulosa cell apoptosis, miR-423, *NORHA*, *SMAD7*, Sow fertility traits

## Abstract

**Background:**

Atresia and degeneration, a follicular developmental fate that reduces female fertility and is triggered by granulosa cell (GC) apoptosis, have been induced by dozens of miRNAs. Here, we report a miRNA, miR-423, that inhibits the initiation of follicular atresia (FA), and early apoptosis of GCs.

**Results:**

We showed that miR-423 was down-regulated during sow FA, and its levels in follicles were negatively correlated with the GC density and the P4/E2 ratio in the follicular fluid in vivo. The in vitro gain-of-function experiments revealed that miR-423 suppresses cell apoptosis, especially early apoptosis in GCs. Mechanically speaking, the miR-423 targets and interacts with the 3'-UTR of the porcine *SMAD7* gene, which encodes an apoptosis-inducing factor in GCs, and represses its expression and pro-apoptotic function. Interestingly, FA and the GC apoptosis-related lncRNA *NORHA* was demonstrated as a ceRNA of miR-423. Additionally, we showed that a single base deletion/insertion in the miR-423 promoter is significantly associated with the number of stillbirths (NSB) trait of sows.

**Conclusion:**

These results demonstrate that miR-423 is a small molecule for inhibiting FA initiation and GC early apoptosis, suggesting that treating with miR-423 may be a novel approach for inhibiting FA initiation and improving female fertility.

**Supplementary Information:**

The online version contains supplementary material available at 10.1186/s40104-023-00960-y.

## Introduction

MicroRNAs (miRNAs) are 18–23 nucleotides (nt) long, single-stranded, non-coding RNAs (ncRNAs) that are generated endogenously. miRNAs usually destabilise target mRNAs or suppress target mRNA translation by interacting with their seed sequences and the miRNA recognition elements (MREs) in the 3′-UTR of target mRNAs by entering the miRNA-induced silencing complexes (miRISCs) [1, 2]. miRNAs are distributed in almost all organs, tissues, and cells and in various organelles, such as mitochondria and exosomes, and regulate an organism’s development, tissue and organ formation, cellular status, and organelle functions [3–5]. Functional disorders of miRNAs often lead to developmental abnormalities, aging, and diseases [6, 7]. Although miRNAs were the first ncRNAs to be discovered, they are still the most studied because of their wide distribution, conserved sequences, simultaneous actions on multiple target mRNAs, and crucial functions [2, 4, 8]. Additionally, the main mechanism of action of other popular ncRNAs, such as circular RNAs (circRNAs) [5, 9, 10], involves acting as competing endogenous RNAs (ceRNAs) of miRNAs.

miR-423 is an intronic miRNA transcribed from the first intron of the protein-coding gene nuclear speckle splicing regulatory protein 1 (*NSRP1*), which encodes a splicing regulator [11]. miR-423 is ubiquitously expressed in various tissues and cells in mammals. It is a multifunctional small molecule and biomarker that contributes to various biological processes related to health and diseases [12–15]. In female mammals, miR-423 is involved in multiple reproductive system functions [16]. For instance, in the uterus, miR-423 participates in preventing trophoblast cell progression, influencing decidualization, and maintaining early pregnancy [17, 18]. In the ovary, miR-423 is associated with some ovarian functions such as 17β-estradiol (E2) synthesis by granulosa cells (GCs) [16], and its dysfunction can lead to ovarian diseases, such as polycystic ovary syndrome and ovarian hyper response [19–21]. We previously found that miR-423 is decreased during follicular atresia (FA) in sows using RNA-seq (unpublished). FA is a follicular developmental fate that reduces female fertility and is triggered by GC apoptosis. In the present study, we aimed to determine the role of miR-423 in GC apoptosis in sows and its mechanism of action.

## Materials and methods

### Animals

Ear tissues of 369 Yorkshire sows (Kangle, Changzhou, China) were obtained for extracting DNA. Data on fertility traits such as the total number of piglets born (TNB), the total number of piglets born alive (NBA), the number of stillbirths (NSB), and the litter weight (LW) were recorded for association analysis. Besides, commercial sow ovaries were obtained for collecting follicles, GCs, and follicular fluid.

### Follicle collection and classification

The follicles (3–5 mm) were collected from sow ovaries and classified into healthy follicles (HF) and atretic follicles (AF) group. Translucent follicles with abundant vascularization, GC number < 4,000/mL, and the ratio of progesterone (P4) (ng/mL) and 17β-estradiol (E2) (pg/mL) levels (that is, the P4/E2 ratio) < 1.0 are classified as HF, others are classified as AF. GC number was counted using a cell counting plate (Qiujing, Shanghai, China). P4 and E2 levels were measured by radioimmunoassay using a P4[I^125^] and E2 Radioimmunoassay Kit (North Pharmaceutical, Beijing, China).

### Quantitative real-time PCR (qPCR)

Trizol (Vazyme, Nanjing, China) was employed to prepare total RNA. cDNA was synthesized from 1 μg of total RNA using HiScript IIQ Select RT SuperMix (Vazyme) or HiScript III 1st Strand cDNA Synthesis Kit (Vazyme). A SYBR Green Master Mix Kit (Vazyme) was employed to quantify the relative transcription levels. The internal references of coding and non-coding genes are *GAPDH* and *U6*, respectively. Primers are shown in Table S1.

### Cell culture and transfection

GCs were obtained from antral follicles (3–5 mm) of mature commercial sows (Zhushun, Nanjing, China). Cell culture and treatment were performed as described previously [5]. DMEM/F12 medium (Gibco, CA, USA) containing 15% fetal bovine serum (FBS) (Gibco) and RPMI-1640 medium (Hyclone, UT, USA) containing 10% FBS and 1% PS were used for culturing sow GCs, and KGN cells, a human GC line. After the cell density reached 80%–90%, Highgene reagent (ABclonal, Wuhan, China), plasmids, and mimics (10 μmol/L) were mixed in 200 μL of medium, and added into cells. Oligonucleotides were prepared by GenePharma (Shanghai, China), and shown in Table S2.

### Apoptosis analysis

An Apoptosis Detection Kit (Vazyme) was employed to measure the apoptosis rate in a flow cytometer (Becton Dickinson, NJ, USA). FlowJo v7.6 software (Stanford University, USA) was employed to analyze data of apoptosis rate.

### Bioinformatics analysis

The nucleotide sequences are from the Ensembl database (https://www.ensembl.org/index.html). TargetScan (https://www.targetscan.org), miRDB (http://www.mirdb.org), and TarBase (https://dianalab.e-ce.uth.gr/html/diana/web/index.php?r=tarbasev8) were employed to search targets of miR-423. RNAhybrid (https://bibiserv.cebitec.uni-bielefeld.de/rnahybrid) was employed to discover the MREs of miR-423. Gene Ontology (GO) and Kyoto Encyclopedia of Genes and Genomes (KEGG) analysis was conducted by Kobas (http://bioinfo.org/kobasrg).

### Subcellular localization

Nucleoplasmic separation of GCs was performed as described previously [5]. miR-423 levels in nuclear and cytoplasmic products were quantified by qPCR, and nuclear and cytoplasmic markers were *GAPDH* and *U6*, respectively.

### Plasmids and luciferase assay

Plasmids include the pmirGLO vectors of *SMAD7* 3'UTR and *NORHA* harboring the MRE of miR-423 and its mutant version that were prepared by Tsingke (Nanjing, China), and the pcDNA3.1-*SMAD7* and pcDNA3.1-*NORHA* vectors that were previously prepared [5, 22]. A Dual-Luciferase Reporter Assay System (Vazyme) was employed for luciferase assay.

### Western blot

Total protein of GCs was obtained by RIPA lysis buffer (Biowrld, MN, USA) containing 1% PMSF (Beyotime, Nantong, China) and protease inhibitor. After detecting concentration by using a BCA Kit (Biosharp, Beijing, China), proteins were electrophoretically separated by 4%–20% SDS-PAGE and transferred to polyvinylidene fluoride membranes (Millipore, Billerica, USA). To prevent non-specific binding, the membranes were blocked with 5% BSA for 1.5 h and then incubated with primary antibody overnight at 4 °C. After incubating the second antibody for 1 h, the ECL chromogenic agent (Vazyme, Nanjing, China) was used to obtain high-resolution images in Amersham ImageQuant 800 system (Cytiva, Japan). Serve as an internal control, anti-GAPDH antibody (1:1,000 dilution) was obtained from Sangon (Shanghai, China). Other primary antibodies including anti-SMAD7 and anti-p-SMAD3 were obtained from Sangon and diluted at 1:1,000.

### RNA pull-down assay

This assay was conducted as described previously [5]. C-1 magnetic beads were obtained from Life Technologies (USA). The probe labeled with biotin is carried out by Biotech (Shanghai, China), and its sequence is 5'-TTT TTT CGG GTC TGT AAT GC-3'. The RNeasy Mini Kit (QIAGEN, Germany) was employed for isolating RNA from the elution for quantifying miR-423 by qPCR.

### Variant identification and association analysis

Three hundred and sixty-nine DNAs were isolated using a phenol–chloroform method and were used as the template of PCR amplification for Sanger sequencing (Sangon). Primers are listed in Table S1. The genotype of each individual is determined using Chromas v2.3 software (https://technelysium.com.au/wp/chromas) based on the sequencing peak diagram. Association analyses were performed using a general linear model as previously described [23].

### Statistics

Unpaired *t*-test and one-way analysis of variance of Prism v8.3 software (GraphPad, USA) were employed for statistical analysis. Multiple comparisons were conducted using a Tukey method.

## Results

### miR-423 is involved in sow FA

**Fig. 1:**
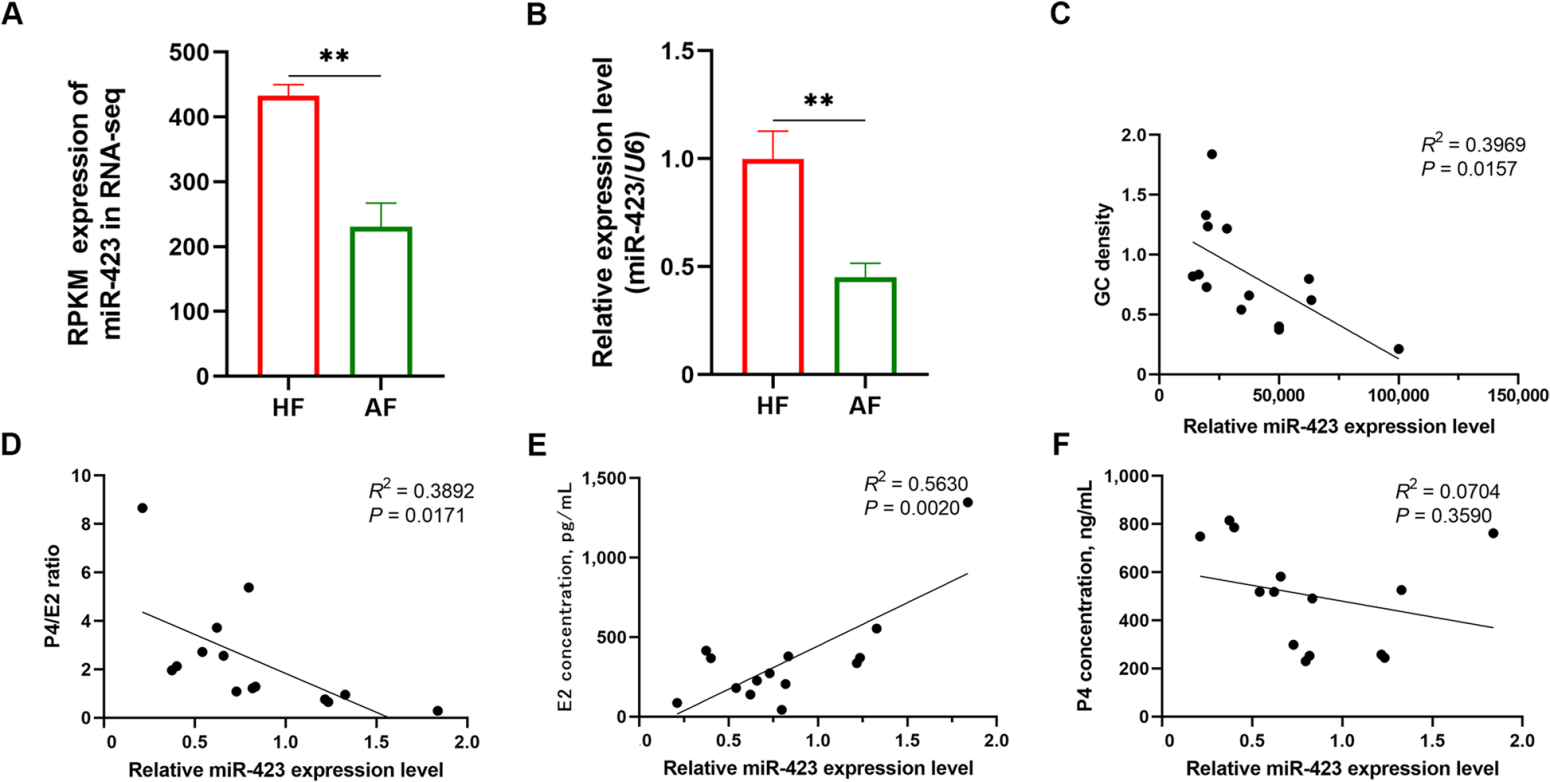
miR-423 is strongly involved in sow follicular atresia. **A** and **B** miR-423 was markedly decreased in sow atretic follicles. **A** RNA-seq data. **B** qPCR. HF, healthy follicles; AF, atretic follicles. **C–F** Correlation analysis. Sow follicles and follicular fluid were collected, and the GC density in follicular fluid was detected using a cell counting plate, E2 and P4 concentration were measured by ELISA, and correlation analysis between miR-423 levels and the GC density (**C**), the P4/E2 ratio (**D**), E2 concentration (**E**), or P4 concentration (**F**) were carried out, respectively. Data represented are mean ± SEM for at last three independent experiments. ^**^P < 0.01

Our previous RNA-seq results revealed that miR-423 expression is markedly decreased in the atretic follicles of sows (Fig. 1A). Further, it was confirmed using qPCR that atretic follicles of sows have significantly lower miR-423 levels than healthy follicles (Fig. 1B), indicating that miR-423 may be involved in the development of FA in sows. Next, we investigated the relationship between miR-423 and sow FA using in vivo experiments. Follicular miR-423 levels were negatively correlated with GC density and the P4/E2 ratio, two core biomarkers of FA in the follicular fluid (Fig. 1C–D). Additionally, follicular miR-423 levels were markedly positively correlated with E2 concentration but not with P4 concentration in the follicular fluid (Fig. 1E–F). Taken together, these results support our hypothesis that miR-423 is directly associated with sow FA.

### miR-423 represses GC apoptosis

**Fig. 2:**
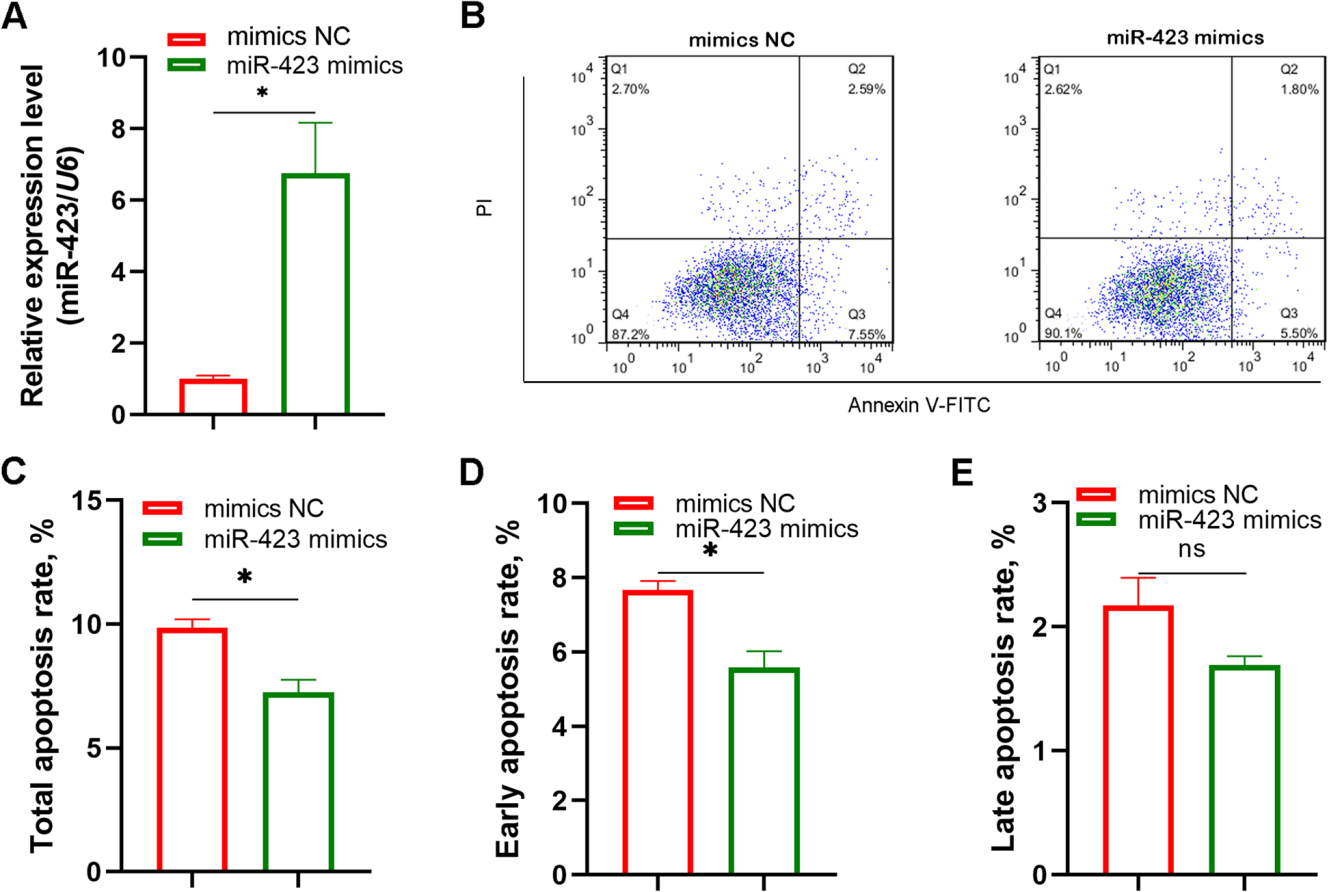
miR-423 represses GC apoptosis. **A** Overexpression efficiency assay. GCs were transfected with miR-423-specific mimics, miR-423 level was detected by qPCR. **B–E** Detection of GC apoptosis rate. Q1, injured or dead cells. Q2, early apoptosis cells. Q3, live cells. Q4, late apoptosis cells. GCs were transfected with miR-423-specific mimics, apoptosis was detected by flow cytometry (**B**), and total apoptosis rate (**C**), early apoptosis rate (**D**) and late apoptosis rate (**E**) was calculated, respectively. NC, negative control. Data represented are mean ± SEM for three independent experiments. ^*^P < 0.05. ns, not significant

GC apoptosis is the fundamental cause of FA in sows [23]. To confirm the role of miR-423 in GC apoptosis, we first treated GCs with miR-423-specific mimics to increase the miR-423 levels (Fig. 2A). Flow cytometry showed that the cell apoptosis rate was considerably reduced in GCs treated with miR-423-specific mimics (Fig. 2B), indicating that miR-423 is an anti-apoptotic miRNA in GCs. Interestingly, the early and not late apoptosis rates were markedly declined in miR-423-treated GCs (Fig. 2C–E). These data suggest that miR-423 is a key modulator of early GC apoptosis and FA initiation in sows.

### *SMAD*7 is a potential target of miR-423

**Fig. 3:**
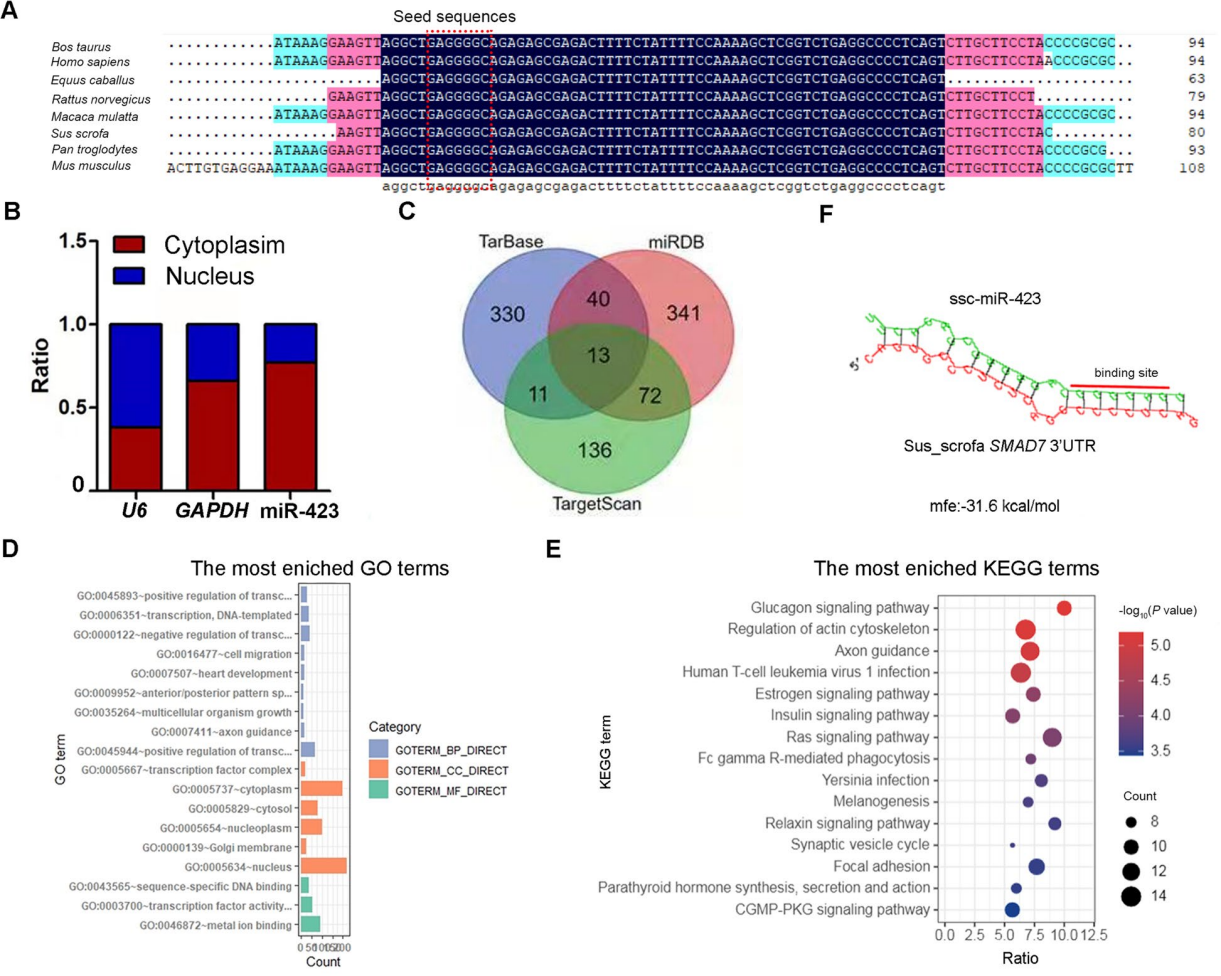
*SMAD7* is a potential target of miR-423. **A** Alignments of pre-miR-423 sequences in mammals. **B** Subcellular distribution of miR-423 in GCs. The levels of internal reference genes (*U6* in the nucleus and *GAPDH* in the cytoplasm) and miR-423 were detected by qPCR, respectively. **C–E** Potential function prediction of miR-423. The potential targets of miR-423 were predicted by three algorithms (TarBase, miRDB, TargetScan) (**C**), GO (**D**) and KEGG (**E**) analysis with miR-423 targets were performed, respectively. **F** The minimal free energy (mfe) between miR-423 and the 3'-UTR of the porcine *SMAD7* gene was predicted by RNAhybrid. The heatmap shows the −log_10_ (*P* value) of the terms. Data represented are mean for three independent experiments

Next, we characterised the porcine miR-423, ssc-miR-423, transcribed from intron 1 of the protein-encoding gene *NSRP1* on pig chromosome 12 (Fig. S1). The precursor sequence of ssc-miR-423 is 80 bp long and is highly homologous with the precursor sequence of other vertebrate miR-423, particularly the mature and seed sequences (Fig. 3A). Subcellular localization analysis showed that miR-423 mainly exists in the cytoplasm of sow GCs (Fig. 3B), indicating that miR-423 suppresses GC apoptosis, possibly through an RNA interference mechanism. Based on this assumption, we employed three algorithms to predict the potential targets of miR-423 with putative MREs in the 3′-UTR. A total of 943 potential targets were identified (Fig. 3C), which were significantly enriched in FA- and GC apoptosis-related signaling pathways, such as the oestrogen, insulin, and Ras signaling pathways (Fig. 3D–E). Interestingly, among the 13 common potential targets observed, *SMAD7* is a well-known inductor of sow GC apoptosis [22, 24]. RNAhybrid prediction showed that miR-423 has a strong binding ability to the porcine *SMAD7* 3′-UTR (Fig. 3F), indicating that *SMAD7* is a potential target of miR-423 in swine.

### *SMAD7* directly targets miR-423 in GCs

**Fig. 4:**
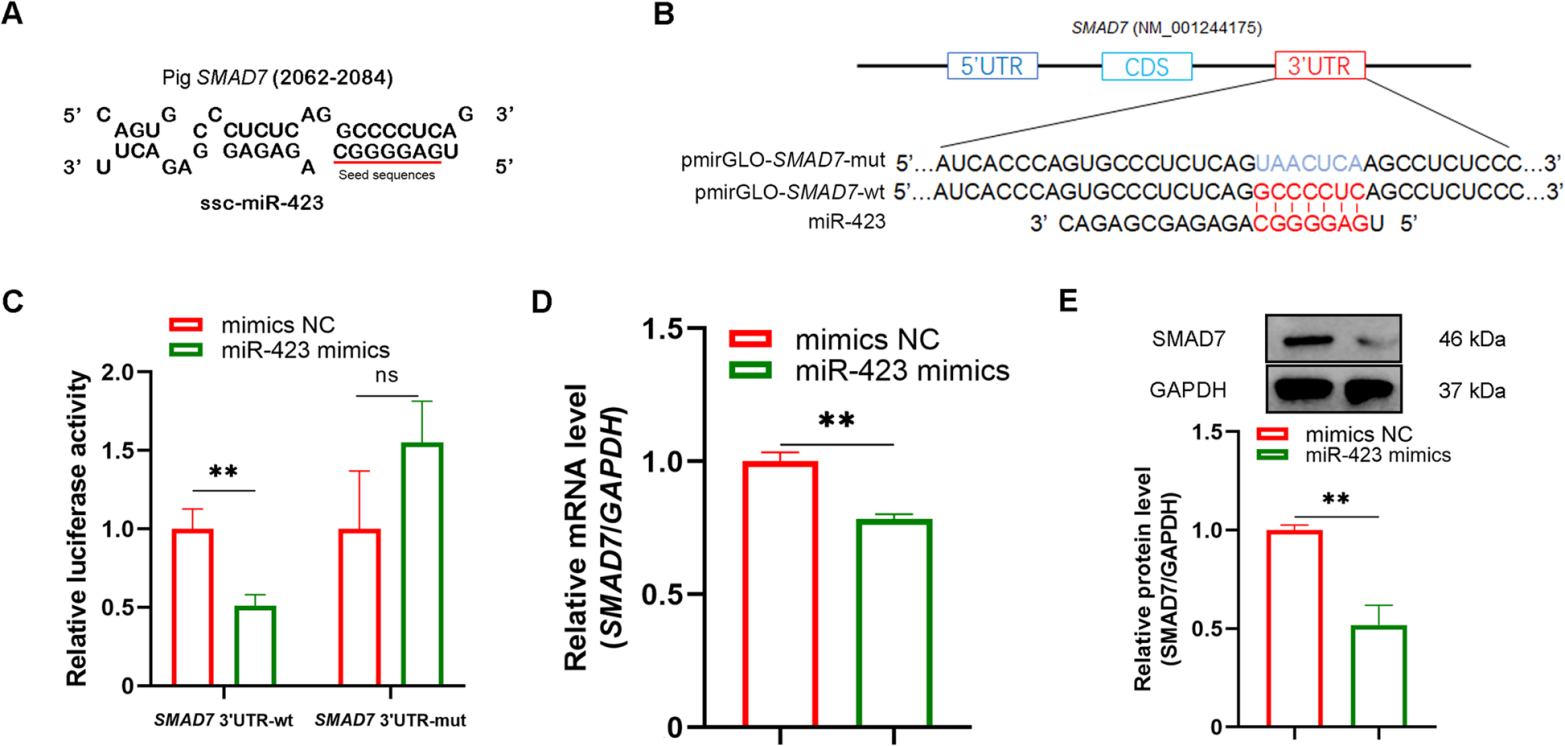
*SMAD7* is a direct target of miR-423 in GCs. **A** Schematic showing the MRE of miR-423 in the 3'UTR of the porcine *SMAD7* gene. **B** Schematic annotation of the reporter vectors of *SMAD7* 3'UTR with wild-type and mutated MRE of miR-423. **C** Luciferase assay. GCs were co-transfected with the reporter vectors and miR-423-specific mimics, luciferase activity was determined. **D–E** miR-423 regulates SMAD7 levels in GCs. GCs were transfected with miR-423-specific mimics, qPCR and western blotting were performed to detect the levels of SMAD7 mRNA (**D**) and protein (**E**). wt, wild type; mut, mutation; NC, negative control. Data represented are mean ± SEM for three independent experiments. ^**^P < 0.01

An MRE of miR-423 was discovered at 2,062–2,084 nt in the porcine *SMAD7* 3'-UTR (NM_001244175), which is fully complementary to the seed sequence of miR-423 (Fig. 4A). To determine whether miR-423 targets *SMAD7*, two reporter vectors of *SMAD7* 3′-UTR were prepared (Fig. 4B). Co-transfection assay with miR-423 mimics revealed that miR-423 induced downregulation of the reporter vector of *SMAD7* 3′-UTR, whereas it had no effect on the reporter with mutated MRE of miR-423 (Fig. 4C), suggesting that *SMAD7* is a direct target of miR-423. Compared with the control group, miR-423-treated sow GCs had significantly diminished SMAD7 levels (Fig. 4D and E, and Fig. S2), suggesting that miR-423 can directly interact with *SMAD7* in sow GCs.

### miR-423 suppresses GC apoptosis through *SMAD7*

**Fig. 5:**
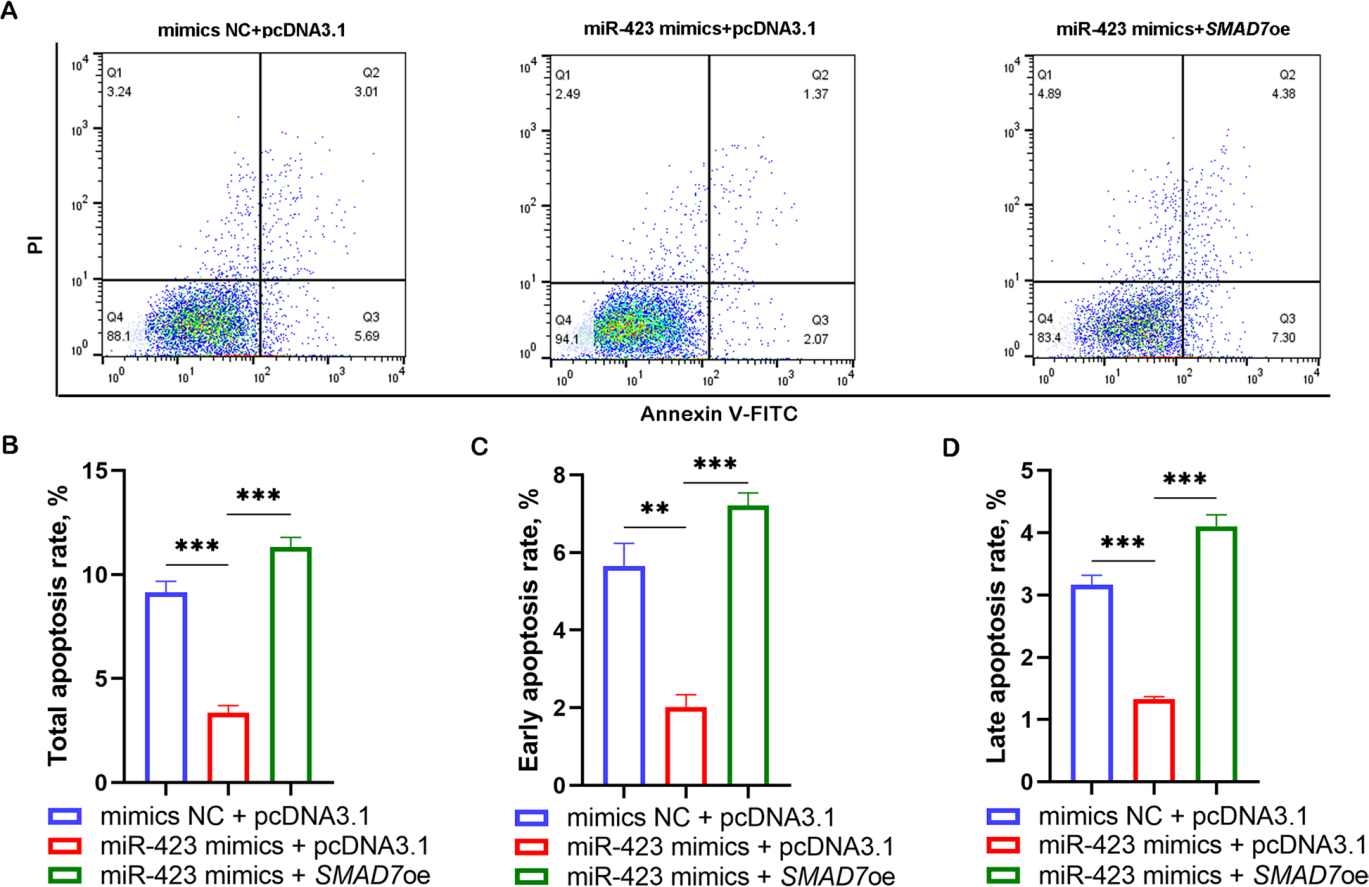
miR-423 regulates GC apoptosis through *SMAD7*. GCs were co-transfected with miR-423-specific mimics and *SMAD7* overexpression vector, and apoptosis was detected by flow cytometry (**A**), and total apoptosis rate (**B**), early apoptosis rate (**C**) and late apoptosis rate (**D**) was calculated, respectively. oe, overexpression; NC, negative control. Data represented are mean ± SEM for three independent experiments. ^***^P < 0.001. ^**^P < 0.01. ^*^P < 0.05

The above results indicated that miR-423 represses GC apoptosis and targets *SMAD7* in GCs. To further determine whether miR-423 supresses GC apoptosis via *SMAD7*, miR-423-specific mimics and *SMAD7* overexpression vector were co-transfected into GCs. Flow cytometry showed that *SMAD7* impeded the function of miR-423 (Fig. 5A), which is inhibition of cell apoptosis, and total apoptosis, early apoptosis, and late apoptosis exhibited the same trend (Fig. 5B–D). This suggested that miR-423 regulates apoptosis by suppressing *SMAD7* expression in sow GCs.

### lncRNA *NORHA* sponges miR-423 in GCs

**Fig. 6:**
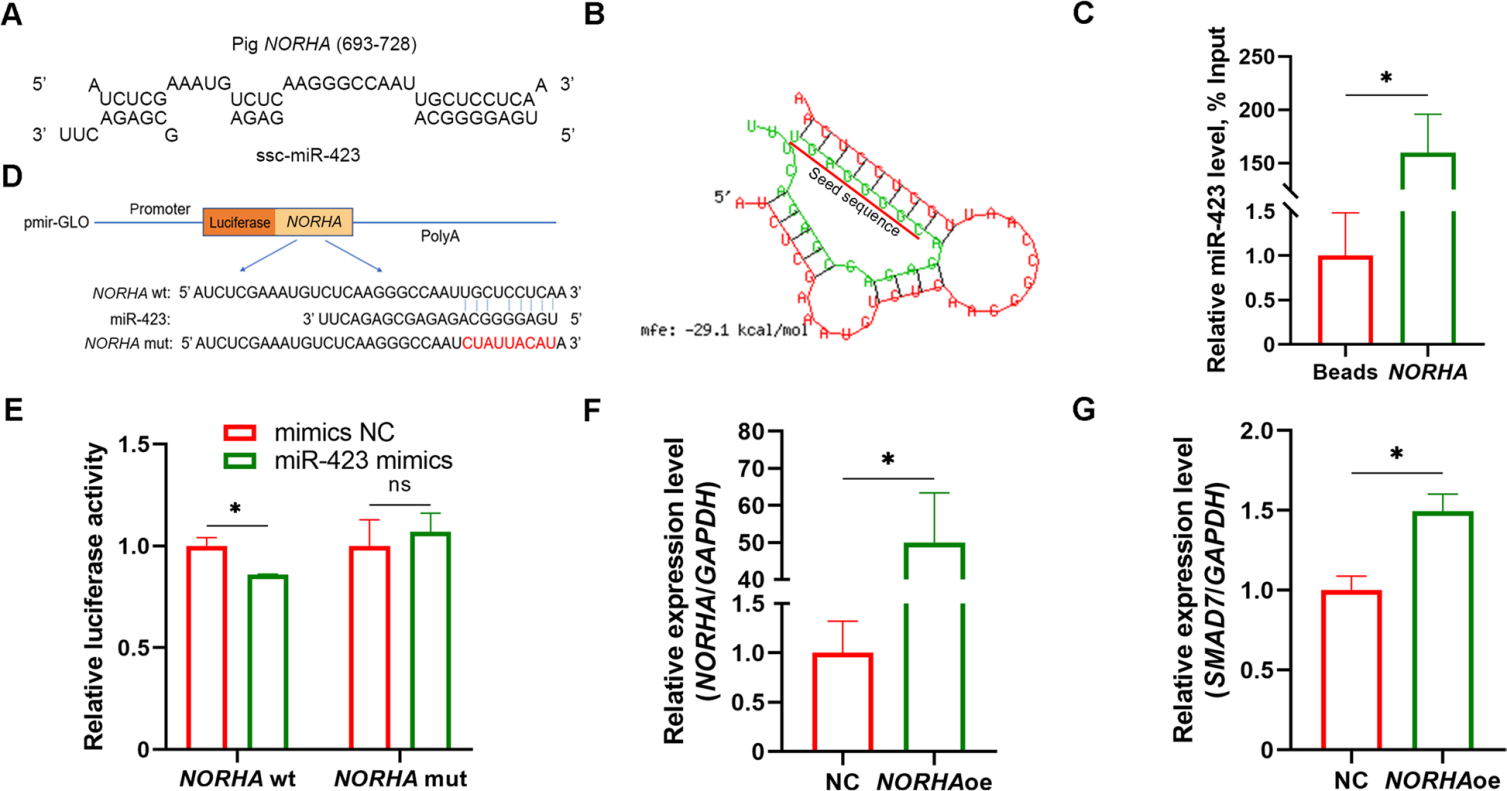
*NORHA* is an ceRNA of miR-423 in GCs. **A** Schematic showing the MRE of miR-423 in the porcine *NORHA* transcript. **B** The minimal free energy (mfe) between miR-423 and *NORHA* tanscript was predicted by RNAhybrid. **C** RNA pull-down analysis of the physical interaction miR-423 and *NORHA* transcript in GCs. **D** Schematic annotation of the reporter vectors of *NORHA* with the wild-type and mutated MRE of miR-423. **E** Luciferase assay. KGN cells were co-transfected with the reporter vectors and miR-423-specific mimics, luciferase activity was determined. **F–G** *NORHA* regulates *SMAD7* expression in GCs. GCs were transfected with the pcDNA3.1-*NORHA*, the levels of *NORHA* (**F**), and *SMAD7* mRNA (**G**) were detected by qPCR. wt, wild type; mut, mutation; NC, negative control. Data represented are mean ± SEM for three independent experiments. ^*^P < 0.05. ns, not significant

Interestingly, an MRE of miR-423 was discovered at 693–728 nt in the porcine *NORHA* transcript (Fig. 6A), a long non-coding RNA (lncRNA) related to sow FA [23]. RNAhybrid demonstrated that miR-423 has a strong binding ability to the porcine *NORHA* transcript (Fig. 6B). To test whether *NORHA* interacts with miR-423 in sow GCs, RNA pull-down experiments were performed using a biotin-labelled *NORHA* probe, and physical interactions between *NORHA* and miR-423 were observed (Fig. 6C). Furthermore, luciferase assay revealed that miR-423 downregulated the reporter of *NORHA* with the wild-type but not the mutated MRE of miR-423 (Fig. 6E), indicating that *NORHA* interacts with miR-423 via its MRE motif. Additionally, *NORHA* suppressed the expression of *SMAD7* targets by miR-423 in GCs (Fig. 6F–G). Taken together, these results suggest that the lncRNA *NORHA* is a ceRNA of miR-423 in sow GCs.

### miR-423 mediated the regulation of *SMAD7* levels and GC apoptosis by *NORHA*

**Fig. 7:**
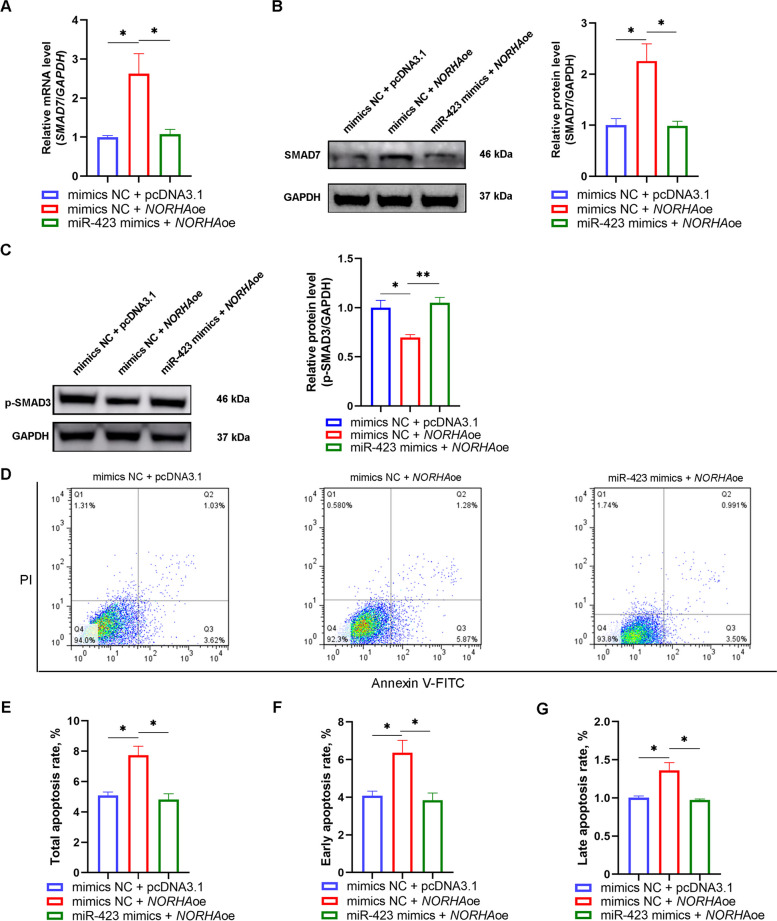
miR-423 mediated *NORHA* regulation of *SMAD7* levels and GC apoptosis. **A–C** GCs were co-transfected with the pcDNA3.1-*NORHA* and miR-423 mimics, and the levels of *SMAD7* mRNA (**A**) were detected by qPCR, and the levels of SMAD7 protein (**B**) and p-SMAD3 protein (**C**) were detected by western blotting. **D–G** GCs were co-transfected with the pcDNA3.1-*NORHA* and miR-423 mimics, and apoptosis was detected by flow cytometry (**D**), and total apoptosis rate (**E**), early apoptosis rate (**F**) and late apoptosis rate (**G**) was calculated, respectively. oe, overexpression; NC, negative control. Data represented are mean ± SEM for three independent experiments. ^*^P < 0.05

Next, we investigated whether miR-423 mediates the promotion of *SMAD7* expression by *NORHA*. Co-transfection with miR-423-specific mimics and the *NORHA* overexpression vector revealed that miR-423 mimics reversed the upregulation of SMAD7 caused by *NORHA* overexpression (Fig. 7A–B, and Fig. S3), indicating that *NORHA* enhanced SAMD7 expression through miR-423 sponging. SMAD7 is an antagonist of the TGF-β pathway; therefore, we investigated whether the activity of the TGF-β pathway is regulated by the *NORHA*/miR-423 axis. *NORHA* significantly diminished the levels of phosphorylated SMAD3 (p-SMAD3), the marker for the activity of the TGF-β pathway; however, this process was reversed by the miR-423 mimics (Fig. 7C and Fig. S4), indicating that *NORHA* inactivates the TGF-β pathway through miR-423 sponging. Both *NORHA* [23] and miR-423 are pro-apoptotic factors in GCs; therefore, we speculated that *NORHA* induces GC apoptosis through miR-423 sponging. As expected, the co-transfection experiment using miR-423-specific mimics and the *NORHA* overexpression vector confirmed that miR-423 repressed *NORHA*-induced GC apoptosis (Fig. 7D–G). These data suggest that *NORHA* induces *SMAD7* expression and cell apoptosis through miR-423 sponging in sow GCs.

### miR-423 is a candidate gene for sow NSB trait

**Fig. 8:**
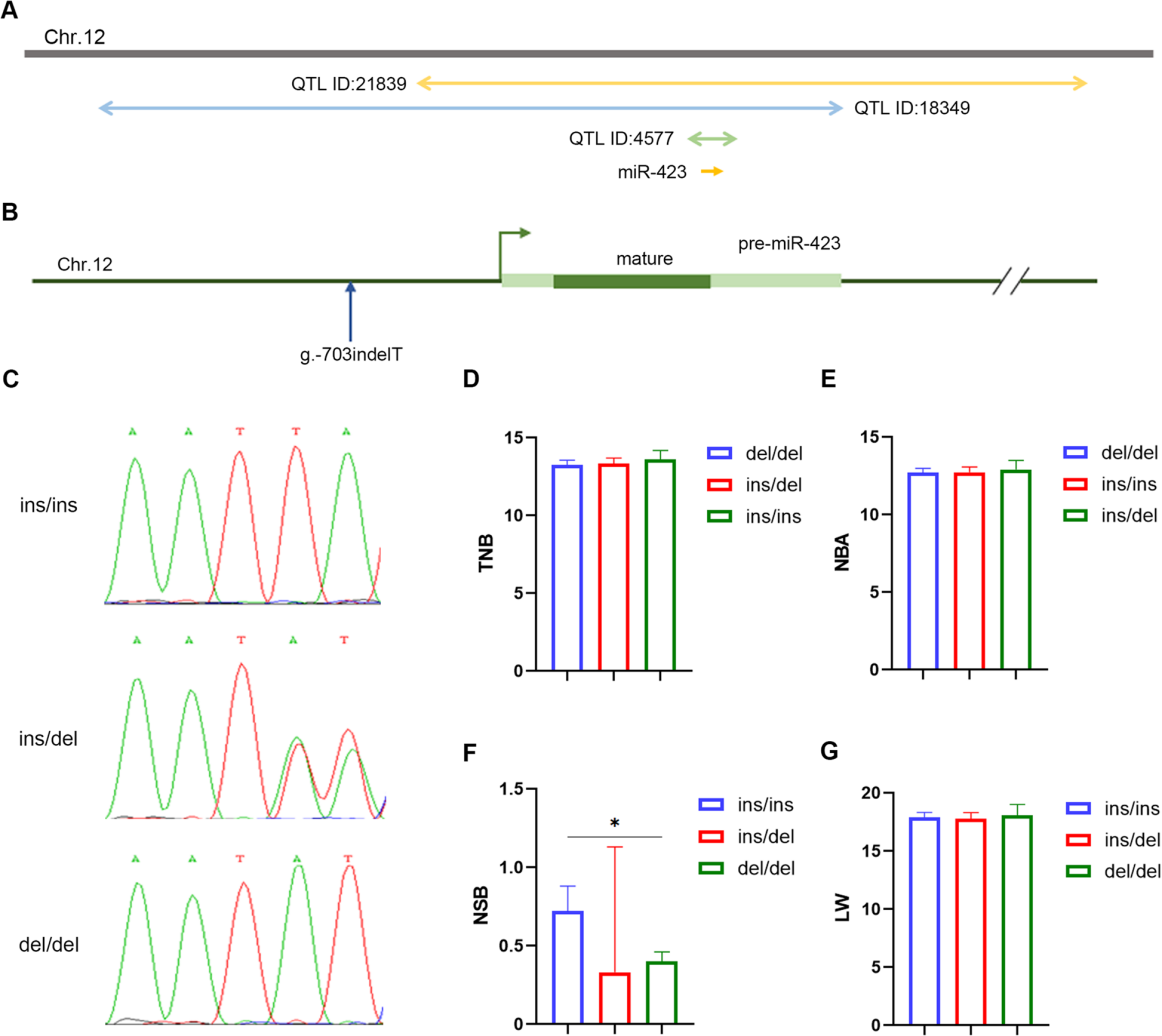
miR-423 is a candidate gene for sow fertility traits. **A** Schematic diagram of the location of miR-423 in QTLs for sow fertility traits. **B** A T ins/del was identified at −703 nt in the promoter of miR-423. **C** Peak plot of three genotypes for the variant g.−703indelT. **D–G** Association analysis between polymorphism of the variant g.−703indelT with the TNB trait (**D**), the NBA trait (**E**), the NSB trait (**F**) and the LW trait (**G**) of Yorkshire sows for primiparity. TNB, the total number of piglets born; NBA, the total number of piglets born alive; NSB, the number of stillbirths; LW, the litter weight. *n* = 369. Data are represented as the least squares means ± SEM. ^*^P < 0.05

miR-423 was located in three QTLs (ID: 4,577, 18,349, and 21,839) for sow fertility traits, which were identified by searching a pig QTL database (Fig. 8A). To ascertain whether miR-423 is a candidate gene for sow fertility traits, we first scanned miR-423 variants using pooled-DNA sequencing. A T ins/del was observed at –703 nt in the promoter, and three genotypes, ins/ins (II), ins/del (ID), and del/del (DD), and two alleles, I and D, were identified in Yorkshire sows (Fig. 8B–D). Association analysis found that the NSB of DD sows was 44.4% (0.32/0.72) lower than that of II sows for primiparity (*P* < 0.05) (Fig. 8E–H). Together, these results suggest that miR-423 is a candidate gene for the NSB trait in Yorkshire sows.

## Discussion

Apoptosis of GCs in follicles is the basic physiological mechanism underlying FA, a condition that affects female fertility. Thus, the mechanism behind GC apoptosis and its regulation have gained increasing attention in the field of female reproduction. GC apoptosis has been reported to be controlled by several factors, including hormones, cytokines, components of the cell death pathway, and ncRNAs like miRNAs, lncRNAs, and circRNAs [25–28]. Among these ncRNAs, miRNAs are widely involved in the regulation of GC apoptosis in various mammals, such as miR-23a and miR-27a in humans [29], miR-126-3p in rats [30], miR-21 in cattle [31], miR-450-5p and miR-202-5p in goats [32], miR-346 in sheep [33], and miR-143 and miR-29c in sows [34, 35]. However, the vast majority of these miRNAs induce GC apoptosis and FA, and only few miRNAs have inhibitory effects and can be used as potential small-molecule regulators to promote follicular development and ovulation and improve female fertility [25, 27]. In this study, we identified a new inhibitor of sow GC apoptosis and FA initiation: miR-423. In other normal cell types such as bone marrow mesenchymal stem cells [36], cardiomyocyte [37], and human umbilical vein endothelial cells [38], miR-423 has been shown to function as an anti-apoptotic modulator. Thus, our study reports a potential small molecule for alleviating FA and improving female fertility. It may even be developed as a potential non-hormonal small-nucleotide drug for treating human ovarian diseases, as it has a highly conserved sequence among mammals.

Generally, miRNAs induce target mRNA decay or prevent target mRNA translation by recognising and interacting with its 3′-UTR through the assemblage of miRISCs with argonaut proteins (AGO1 to AGO4) and other silencing factors [39, 40]. Here, we identified that *SMAD7* is a target of miR-423 in sow GCs and mediates the regulatory effect of miR-423 on the activity of the TGF-β pathway and cell apoptosis. SMAD7 is an inhibitory SMAD protein in the TGF-β pathway, which is strongly related to mammalian ovarian functions including GC states (proliferation or apoptosis) and cycle, secretion of steroid hormones (e.g., E2, P4, and androgen), oocyte-GC communication, and folliculogenesis [22, 41–44]. In Tibetan sheep, miR-21 and let-7b, two miRNAs involved in FA and GC apoptosis, have been found to suppress *SMAD7* expression in GCs [45]. In addition to miR-423, *SMAD7* is a functional target of other miRNAs related to FA and GC apoptosis, such as miR-92a [22], miR-181b [24], and miR-21–5p [44]. miR-424 and miR-503 directly target *SMAD7* to control the proliferation and cycle progression of GCs in bovine [43]. These findings suggest that *SMAD7* is a vital mediator and hub gene of the miRNA network regulating GC functions, FA, and female fertility. Furthermore, our findings report a potential small-molecule activator of the TGF-β pathway for scientific research and clinical practice.

LncRNAs are ncRNAs that are more than 200 nt in length. They often block the inhibition of miRNAs on target mRNAs by acting as ceRNAs of miRNAs in the cytoplasm [10, 46]. *NORHA*, a cytoplasmic lncRNA in sow GCs, is a potential ceRNA of multiple miRNAs that are differentially expressed during sow FA [23]. In this study, we demonstrated that cytoplasmic *NORHA* maintains the levels of *SMAD7* (a target of miR-423) and its function in inducing apoptosis in sow GCs by miR-423 sponging. miR-96, miR-182, miR-183, and miR-187 have been previously confirmed to be sponged by *NORHA* in sow GCs [5, 23]. miR-423 is also sponged by other lncRNAs in other cell types, such as *LINC00680* in oesophageal squamous cell carcinoma cells [46] and *AFAP1-AS1* in nasopharyngeal carcinoma cells [47]. A recent report showed that circRNA-0000081 maintains the levels of *PDPK1* (a target of miR-423) in gastric cancer cells via miR-423 sponging, thereby affecting cell proliferation, migration, and invasion potential [48]. In the present study, we discovered a new signaling pathway, the *NORHA*/miR-423/*SMAD7* pathway, that contributes to FA and GC apoptosis and enriches the ceRNA network of the *NORHA*-miRNAs in GCs.

Increasing evidence indicates that miRNAs also serve as candidate genes for many important traits, including fertility and disease traits [25, 49, 50]; however, they have shorter sequences and relatively fewer variants than protein-encoding genes and lncRNAs. In domestic animals, some miRNA variants are associated with economically important traits. For example, g.17104G > A of miR-208b is associated with the proportions of type I and IIb fibre numbers and drip loss in Berkshire, Landrace, and Yorkshire pigs [51], a G > A variant of miR-22 is related to meat colour parameters in Suhuai pigs [52], g.–113C > A of miR-9 is related to litter size in Markhoz goats [53], and g.–13G > A and g.–24T > G of miR-27a-3p are associated with litter size in Hu sheep [54]. In the present study, we identified a novel variant (g.–703indelT) of miR-423, an inhibitor of sow GC apoptosis, that is markedly associated with the NSB trait. miR-423 is the second miRNA candidate identified to be associated with fertility traits in swine. Recently, miR-23a has also been reported as a candidate miRNA, because its variants (g.–398C > T, g.–283G > C, and g.–271C > T) are associated with sow fertility traits [25, 55]. Additionally, because miR-423 is the first candidate gene identified among the three QTLs (ID: 4,577, 18,349, and 21,839) for sow fertility traits, these QTLs are miRNA-related QTLs (mirQTLs).

## Conclusions

To conclude, we demonstrate that miR-423 is involved in the sow FA initiation by repressing the early apoptosis of GCs. *SMAD7* and *NORHA* was identified as a new target and a new ceRNA of miR-423, and the three form a signaling pathway that controls FA and GC apoptosis (Fig. S5). Importantly, we demonstrate that miR-423 is a new candidate miRNA for sow fertility traits.

These results demonstrate that miR-423 is a potential small molecule for inhibiting FA initiation and GC apoptosis, suggesting that treating with miR-423 may be a novel approach for inhibiting FA initiation and improving female fertility.

### Supplementary Information

**Additional file 1.** **Table S1.** Primers designed for reverse-transcription and qPCR. **Table S2.** Oligonucleotides used in this study.

**Additional file 2. Fig. S1.** Chromosome and genome localization of miR-423 in mammals. **Fig. S2.** Western blot images of miR-423 regulates SMAD7 levels in GCs. **Fig. S3.** Western blot images of miR-423 mediated NORHA regulation of SMAD7 levels in GCs. **Fig. S4.** Western blot images of miR-423 mediated the regulation of p-SMAD3 levels by NORHA in GCs. **Fig. S5.** Working model of miR-423 in sow GCs.

## Data Availability

The datasets used and/or analyzed during the current study are available from the corresponding author on reasonable request.

## Abbreviations

AF: Atretic follicle
ceRNA: Competing endogenous RNA
FA: Follicular atresia
GC: Granulosa cell
HF: Healthy follicle
LW: Litter weight
miRNA: MicroRNA
miRISC: miRNA-induced silencing complexe
MRE: miRNA recognition element
NBA: Total number of piglets born alive
ncRNA: Noncoding RNA
NSB: Number of stillbirths
TNB: Total number of piglets born
