# Supplementary material for: miR-423 sponged by lncRNA NORHA inhibits granulosa cell apoptosis
Source: J Anim Sci Biotechnol. 2023 Dec 5;14:154. doi: 10.1186/s40104-023-00960-y (PMC10696705; doi:10.1186/s40104-023-00960-y)
Supplement: Supplementary file 1 — Additional file 1. Table S1. Primers designed for reverse-transcription and qPCR. Table S2. Oligonucleotides used in this study. [file 40104_2023_960_MOESM1_ESM.docx]

**Additional file 1**

**Table S1** Primers designed for reverse-transcription and qPCR

**Table S2** Oligonucleotides used in this study

**Table S1** Primers designed for reverse-transcription and qPCR

| **Genes** | **Primer sequence (5**' **to 3**'**)** | **Usage** |
| --- | --- | --- |
| *miR-423* | CCTGTTGTCTCCAGCCACAAAAGAGCACAATATATTTCAGGAGACAACAGGAAAGTCT | Reverse transcription |
| *miR-423* | F: CCTGTTGTCTCCAGCCACAAAAGAGCAC | qPCR |
|  | R: AATATTTCAGGAGACAACAGGAAAGTCT |  |
| *miR-423* | F: TTCCTTTCTTCCCACCG | genotyping |
|  | R: AGCCCACTTGCTTTGTCTT |  |
| *SMAD7* | F: TCACGCGGGAAGTGGAT | qPCR |
|  | R: GGCTGTACGCTTTCTCATAGTC |  |
| *U6* | F: GCTTCGGCAGCACATATACT | qPCR |
|  | R: TTCACGAATTTGCGTGTCAT |  |
| *GAPDH* | F: CGTGCGGTTGTGGATCT | qPCR |
|  | R: CTCAGTGTAGCCCAGGAT |  |

**Table S2** Oligonucleotides used in this study

| **Name** | **Sequence (5' to 3')** |
| --- | --- |
| mimics NC | UUCUCCGAACGUGUCACGUTT |
|  | ACGUGACACGUUCGGAGAATT |
| miR-423 mimics | UGAGGGGCAGAGAGCGAGACUUU |
|  | AAAGUCUCGCUCUCUGCCCCUCA |
