# Supplementary material for: miR-423 sponged by lncRNA NORHA inhibits granulosa cell apoptosis
Source: J Anim Sci Biotechnol. 2023 Dec 5;14:154. doi: 10.1186/s40104-023-00960-y (PMC10696705; doi:10.1186/s40104-023-00960-y)
Supplement: Supplementary file 2 — Additional file 2. Fig. S1. Chromosome and genome localization of miR-423 in mammals. Fig. S2. Western blot images of miR-423 regulates SMAD7 levels in GCs. Fig. S3. Western blot images of miR-423 mediated NORHA regulation of SMAD7 levels in GCs. Fig. S4. Western blot images of miR-423 mediated the regulation of p-SMAD3 levels by NORHA in GCs. Fig. S5. Working model of miR-423 in sow GCs. [file 40104_2023_960_MOESM2_ESM.docx]

**Additional file 2**

**Fig. S1** Chromosome and genome localization of miR-423 in mammals

**Fig. S2** Western blot images of miR-423 regulates SMAD7 levels in GCs

**Fig. S3** Western blot images of miR-423 mediated NORHA regulation of SMAD7 levels in GCs

**Fig. S4** Western blot images of miR-423 mediated the regulation of p-SMAD3 levels by *NORHA* in GCs

**Fig. S5** Working model of miR-423 in sow GCs


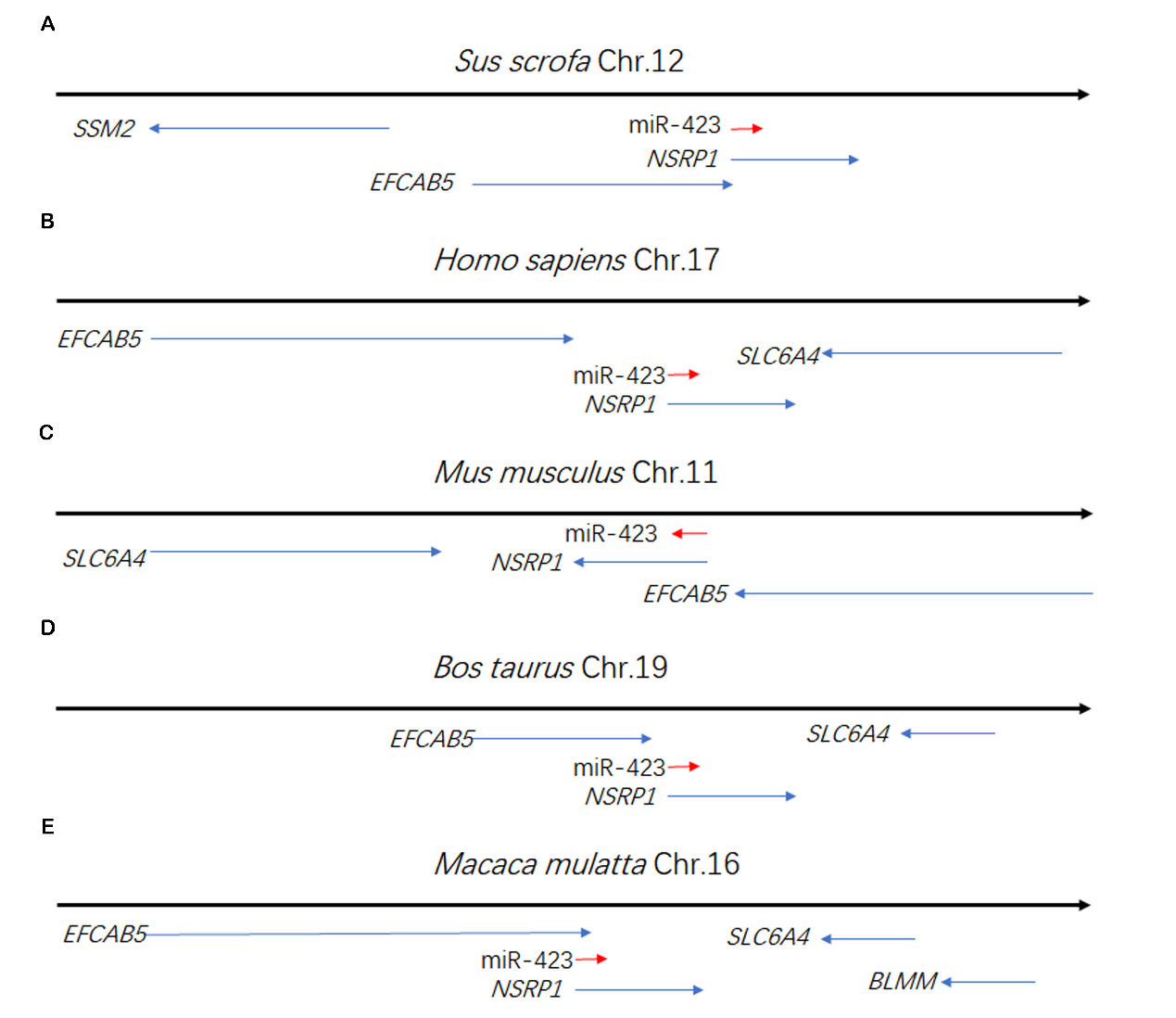


**Fig. S1** Chromosome and genome localization of miR-423 in mammals

SMAD7

**
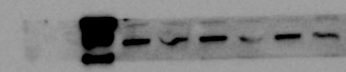
**

GAPDH

**
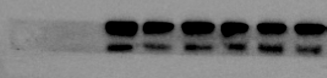
**

**Fig. S2** Western blot images of miR-423 regulates SMAD7 levels in GCs

SMAD7

**
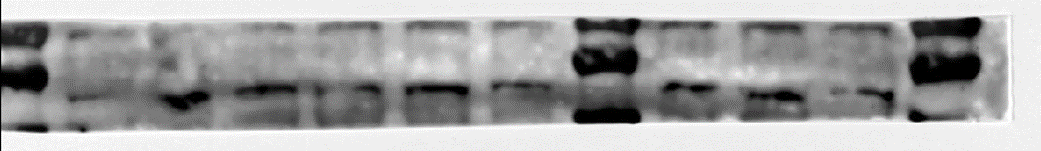
**

GAPDH

**
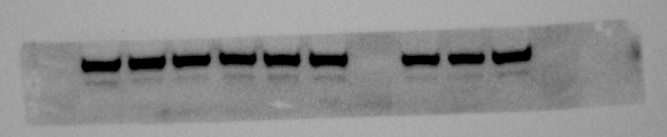
**

**Figure. S3**

**Fig. S3** Western blot images of miR-423 mediated NORHA regulation of SMAD7 levels in GCs

p-SMAD3
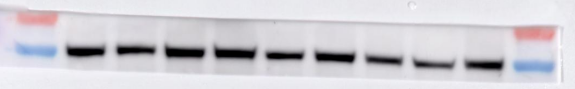


GAPDH


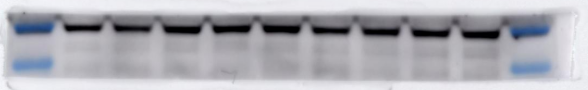


**Fig. S4** Western blot images of miR-423 mediated the regulation of p-SMAD3 levels by *NORHA* in GCs

**
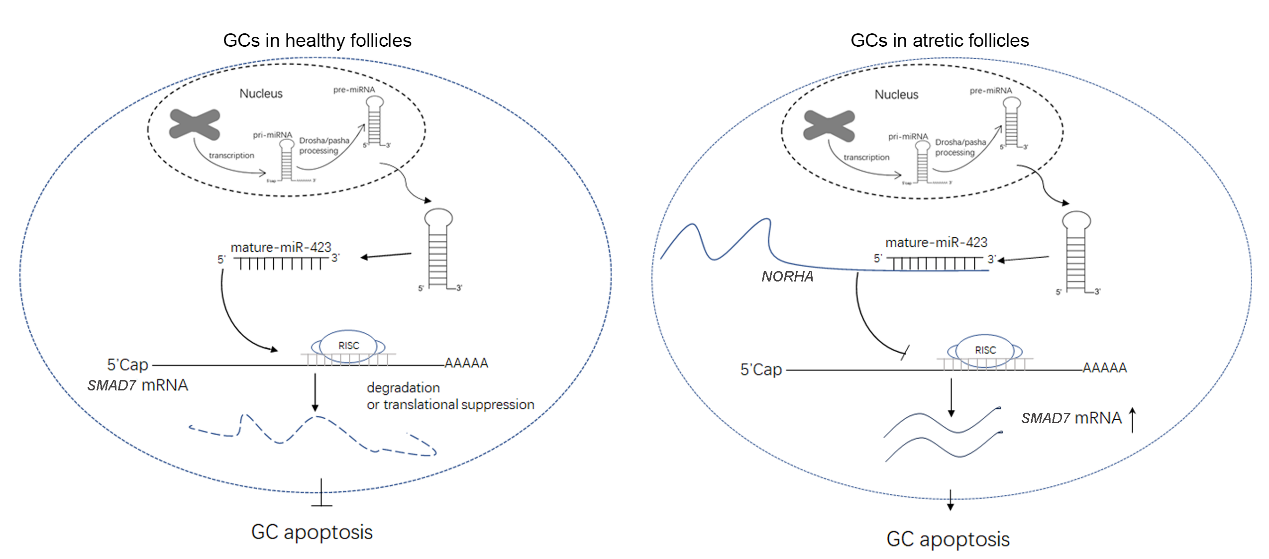
**

**Fig. S5** Working model of miR-423 in sow GCs
